# Errata

**DOI:** 10.1016/j.jacig.2023.100107

**Published:** 2023-04-05

With regard to the article in the February 2022 issue entitled “Blister fluid as a cellular input for *ex vivo* diagnostics in drug-induced severe cutaneous adverse reactions improves sensitivity and explores immunopathogenesis” (J Allergy Clin Immunol Global 2022;1:16-21), the ethical/consent statement appeared in the supplemental materials for the article. This statement is also provided in full below:

Cases with cryogenically stored PBMC and BFC were identified from previous prospective ethically approved studies (PIPA: HREC/15/Austin/75; AUS-SCAR: HREC/50791/Austin-2019, Melbourne, Australia).

With regard to the article in the November 2022 issue entitled “Upregulation of IL-4 receptor signaling pathway in circulating ILC2s from asthma patients” (J Allergy Clin Immunol Global 2022;1:299-304), the ethical/consent statement appeared in the supplemental materials for the article. This statement is also provided in full below:

The study protocol was approved by the Institutional Review Board (IRB) of the Keio University School of Medicine (IRB approval number: 20090009). This study was conducted in compliance with the Declaration of Helsinki. All patients provided written informed consent, and patient anonymity was preserved using methods approved by the IRB.

